# Additive Zirconia in Dentistry: Techniques, Trends, and Future Perspectives

**DOI:** 10.1155/bmri/6602281

**Published:** 2025-10-10

**Authors:** Gunjan Singh Aswal, Renu Rawat, Reisha Rafeek, Dhara Dwivedi, Nitin Prabhakar, Madan Mohan Gupta

**Affiliations:** ^1^ School of Dentistry, Faculty of Medical Sciences, The University of the West Indies, St. Augustine, Trinidad and Tobago, uwi.edu; ^2^ Independent Researcher, Trinidad and Tobago; ^3^ Department of Dental Medicine, College Of Medicine and Health Sciences, Hawassa University, Awasa, Ethiopia, hu.edu.et; ^4^ Department of Pathology, Saveetha Dental College and Hospitals, Saveetha Institute of Medical and Technical Sciences, Saveetha University, Chennai, India, saveetha.com; ^5^ Oral & Maxillofacial Surgery Unit, Department of Dental Medicine, College Of Medicine and Health Sciences, Hawassa University, Awasa, Ethiopia, hu.edu.et; ^6^ School of Pharmacy, Faculty of Medical Sciences, The University of the West Indies, St. Augustine, Trinidad and Tobago, uwi.edu

**Keywords:** 3D printing, additive manufacturing, bioceramics, CAD/CAM, dental prostheses, zirconia

## Abstract

Additive manufacturing (AM) has gained significant traction in the dental field, yet its application in dental ceramics, specifically zirconia (ZrO_2_), is still evolving. ZrO_2_, a widely used biomaterial, has become popular in dental procedures due to its exceptional properties. Although subtractive technologies like milling and CAD/CAM are prevalent for ZrO_2_ restorations, they have limitations. The integration of AM in ceramic restoration production is a burgeoning area of research and industry interest globally, requiring a comprehensive understanding among dental professionals. This review paper explores various AM technologies for ZrO_2_ processing, discussing their advantages and future potential. The results indicate that while techniques like stereolithography and digital light processing can produce ZrO_2_ restorations with improved surface quality and dimensional accuracy, challenges such as porosity, reduced mechanical strength compared to conventional milling, and variability in sintering outcomes persist. The findings show encouraging potential for AM in ZrO_2_‐based restorative, implant, and regenerative dentistry. Despite this, more refinements and substantiation are needed before it can be widely adopted in clinical settings.

## 1. Introduction

Restorative dentistry plays a vital role in the dental field, especially as there is a growing demand for esthetics. A dental restoration repairs or replaces parts of a tooth that have been damaged, bringing back its normal look and use. Various materials, including ceramics, polymers, composites, and metals, have been used as suitable materials for dental restorations [1, 2]. However, the increasing demand for esthetically pleasing restorations has led to metal‐free cores in restorations that match the natural dental elements in color (including hue, chroma, and value).

Polycrystalline ceramics, particularly those utilizing white metallic oxides like alumina and zirconia (ZrO_2_), were developed to replace metal structures without significantly compromising flexural strength [3]. Among these, ZrO_2_ has received substantial attention due to its superior mechanical properties, which stem from its unique morphological structure and chemical composition [4]. The most commonly used ZrO_2_ types in dental applications are yttrium tetragonal ZrO_2_ polycrystals (3Y‐TZPs) and ZrO_2_‐toughened alumina (ZTA). Due to their high flexural strength and fracture toughness, 3Y‐TZPs are commonly used in the fabrication of dental crowns and fixed partial dentures, although their properties are highly sensitive to grain size and sintering conditions. ZTA combines ZrO_2_ with alumina to improve toughness and thermal stability, but exhibits lower mechanical properties than 3Y‐TZP. Recently, ZrO_2_‐containing lithium silicate (ZLS) ceramics have shown to exhibit both strength and translucency, making them a suitable option for monolithic restorations, albeit requiring precise processing to optimize performance [5].

Various conventional techniques such as tape casting, slip casting, and laser cutting, diamond plastic processing, dry pressing, and direct consolidation have been developed for processing and shaping ZrO_2_ ceramics. Additionally, dip coating, extrusion, microwave irradiation, and injection molding have also been explored. While these techniques can produce functional ZrO_2_ parts, there are several limitations, such as extended production time, significant labor and cost demands, rapid wear of machining tools, limited machining precision, and challenges in manufacturing intricate geometries [6].

The advent of computer‐aided design and computer‐aided manufacturing (CAD/CAM) technologies has completely revolutionized dentistry by transforming the design and production of various dental prostheses, including crowns, veneers, inlays, onlays, fixed partial dentures, implant abutments, full mouth reconstructions, and various orthodontic appliances [7]. CAD/CAM, first invented in the 1970s, has streamlined production mainly by increasing production efficiency, accuracy, and reproducibility [8]. Subtractive manufacturing (SM), a commonly used CAD/CAM technique, involves milling designed objects from solid blocks, enabling precise production through computer numeric controlled (CNC) machines. However, this often results in material wastage and limited design complexity.

On the other hand, additive manufacturing (AM), also referred to as 3D printing, has gained recognition in the recent years. It builds objects layer by layer on a 3D model, allowing for intricate designs of dental components and minimizing material wastage [9, 10].

This review paper is aimed at systematically examining the spectrum of AM technologies applicable to ZrO_2_ fabrication, outlining their current capabilities and challenges, and proposing specific avenues for future research. Unlike prior reviews, it highlights specific material and process challenges, integrates economic considerations, and proposes clear, targeted strategies to bridge laboratory research with clinical application.

## 2. Methodology

The present comprehensive review was conducted to evaluate and find collective current literature on the application of AM technologies for processing ZrO_2_ in dental restorations and to understand the future aspects.

An electronic search was done on various databases like PubMed, Scopus, Medline, and Web of Science using keywords: (“Additive Manufacturing” OR “3D Printing” OR “Three Dimensional Printing”) AND (“Zirconia” OR “Zirconium dioxide” OR “ZrO2” OR “Zirconia‐containing ceramics”) AND (“Dental” OR “Dentistry” OR “Dental Restoration” OR “Dental Prosthesis” OR “Dental Crown” OR “Dental Bridge” OR “Dental Implant” OR “Prosthodontics” OR “Restorative Dentistry” OR “Dental Application”), and the studies were included based on the following criteria:

• Peer‐reviewed articles published between January 2010 and December 2024.
• Studies focusing on AM techniques specifically used for ZrO_2_ processing.
• English language articles.
• In vitro, in vivo, and clinical studies, as well as relevant reviews and technical reports.

Studies were excluded if they focused on non‐ZrO_2_ ceramics, discussed AM in dentistry without reference to ZrO_2_, and were non‐English publications and editorials.

## 3. Discussion

### 3.1. AM

Ceramic AM technology has shown promising results in giving high‐precision dental restorations. Its rapid technological progress has notably improved both the accuracy of the printing process and the mechanical integrity of the resulting structures. This, in turn, has resulted in improved accuracy of dental restorations, thereby gaining popularity over the SM technique [11]. Conventional subtractive technologies include various time‐consuming measures (such as prototyping, tooling, and setup), whereas AM permits for quicker direct production beginning with a 3D scan of the oral cavity, thereby reducing the potential for human error and also minimizing the manufacturing steps [12].

According to ISO/ASTM 17296 standard, AM processes can be classified into two main types:

• “Direct” single‐step processes—where components are quickly manufactured in a single process, achieving both the basic dimensions and material attributes for the desired item.
• “Indirect” multistep processes—where components are fabricated through a series of steps.

**Figure 1:**
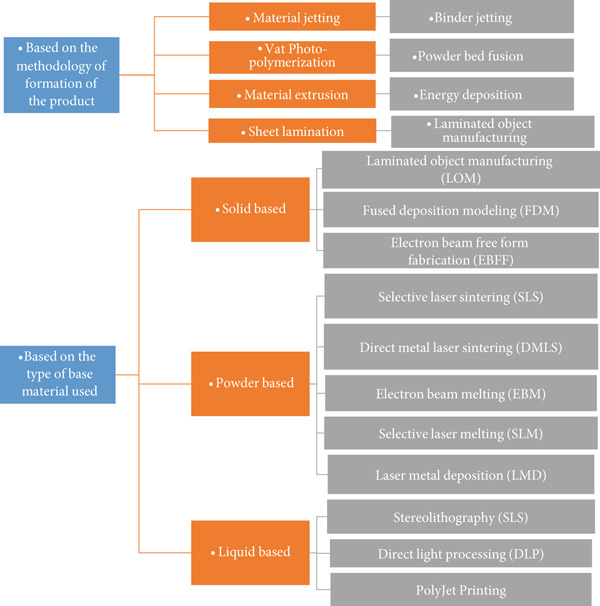
Additive manufacturing techniques.

Typically, all AM techniques follow the fundamental concept of layer‐by‐layer material deposition [6, 13]. Figure 1 presents various types of AM techniques classified based on the method of formation, the type of base material used, and the mechanism of processing.

### 3.2. AM Techniques

*Vat photopolymerization* includes stereolithography (SLA) and digital light processing (DLP). This light‐activated polymerization method selectively cures liquid photopolymer mixed with ceramic resin using light. SLA, which uses a laser to cure resin layer by layer in a vat, was developed in 1986 by Chuck Hull and became widely used in medicine, particularly for creating surgical models. The process involves curing resin mixed with ceramic powder using light, either from a laser or LED, in a vat. A preprogrammed UV light beam outlines each layer of the object, and subsequent layers are built until the final part is formed [14]. The method offers precision and enhanced surface quality, enabling intricate shapes without high‐energy lasers, making SLA particularly useful for ZrO_2_ devices. Benefits of SLA include rapid production, high surface quality, and minimal raw material consumption. However, SLA requires support structures and has limited scalability for mass production. SLA‐fabricated ZrO_2_ exhibits a Vickers hardness of 1398 HV and a flexural strength of 200.14 MPa. The associated surface roughness is around 2.06 *μ*m [15, 16]. These crowns exhibit satisfactory trueness, showing minimal deviation from the intended design, and exhibit excellent fracture resistance, which ensures their ability to withstand the mechanical stresses and forces encountered in the oral environment, making them a reliable option for dental applications [17, 18]. DLP, developed by Larry Hornbeck in 1987, is similar to SLA but has a different light source. It uses a digital micromirror device (DMD) to project light across an entire layer simultaneously, thereby enabling faster printing [19]. ZrO_2_ produced via DLP exhibits high densification and excellent optical transmittance, with a flexural strength of 831 ± 74 MPa, therefore being suitable for dental applications [20].

*Material jetting/inkjet printing* involves selectively depositing UV‐polymerizable polymers, which are cured by UV light based on virtual designs. This method allows precise material deposition, enabling customization of color, material properties, and spatially graded structures. Inkjet printing can be indirect, where binder droplets are injected into a powder layer, or direct, where material is deposited directly via an inkjet head [21]. Direct inkjet printing is widely used for dense, high‐accuracy ceramic objects with complex shapes, minimizing material usage. In this process, ceramic powder suspension is applied onto a substrate from a nozzle, forming solid portions as droplets are deposited and undergo phase transitions. The ceramic ink is vaporized and guided by computer data, allowing layer‐by‐layer construction of 3D ceramic parts [22]. Inkjet printing effectiveness depends on ceramic particles and ink formulation, in addition to properties like flow resistance and surface tension. A common issue, known as “coffee staining,” occurs when solid particles separate during drying, causing defects. This can be minimized by adding 10% polyethylene glycol to the ink formulation [23]. ZrO_2_ suspension can be printed using a multinozzle printer to create vertical walls with a thickness of approximately 340 *μ*m and a spacing of 350 *μ*m. Alignment in the x‐direction is more accurate than in the y‐direction, with walls in the y‐direction having sharp edges and those in the x‐direction being rounded. Additionally, despite a nominal wall height of 3 mm, the wall height can vary [24].

*Fused deposition modeling* (FDM), or material extrusion, heats ceramic material beyond its melting point and extrudes it through a nozzle layer by layer, enabling precise 3D component creation. The material is fed through a heating nozzle, and the platform moves vertically, forming objects with thermoplastics. FDM allows for the use of multiple materials simultaneously, offering cost‐effective production of high‐strength, moisture‐resistant materials in various colors. However, it has limitations, including weak mechanical properties, visible layer lines, and limited thermoplastic options. Postproduction processes like debinding and sintering may be required to address these issues, and temperature fluctuations may necessitate supporting materials [13, 14]. *Direct ink writing* (DIW), or robocasting, uses nozzles to deposit high‐solid ceramic suspensions layer by layer. After deposition, debinding and sintering processes eliminate organic materials. DIW allows for high‐resolution control over porosity and structural design, making it ideal for scaffold production and tissue engineering. Key factors influencing product quality include material viscosity, nozzle size, scanning speed, and drying steps. Managing slurry flocculation and incorporating gelling agents, binders, and plasticizers optimize deposition for intricate 3D structures [25]. *Binder jetting* (BJ) forms objects by bonding powder particles with a liquid binder, building them layer by layer without heat, preventing residual stresses. BJ is efficient for printing large ceramic objects and is economical than other methods. However, it faces challenges such as high porosity, which can result in weaker mechanical properties. Infiltrating the printed object with a glass material under vacuum can improve density and reduce porosity [26].

*Powder-based printing methods*, including direct metal laser sintering (DMLS), selective laser sintering (SLS), selective laser melting (SLM), and electron beam melting (EBM), involve fusing powdered materials using different energy sources, such as lasers or electron beams. While SLS, DMLS, and SLM use lasers, EBM employs an electron beam to fuse the material [27]. In SLS, a high‐power laser sinters ceramic powder in layers to create 3D objects, with no need for support structures, as loose powder supports overhangs. There are two types of SLS: direct, where ceramic particles are sintered directly, and indirect, where a binder is used before sintering. Challenges with SLS include difficulties in densification and thermal stresses that can cause cracking, especially with high‐melting ceramics like ZrO_2_ [28]. DMLS uses laser beams to melt metal powders into durable materials, offering high accuracy and complex shapes but facing potential issues with porosity and material degradation [13]. SLM fully melts metal powders, preventing porous structures and resulting in better material bonding and mechanical properties. However, SLM can cause internal tension due to rapid heating and cooling, necessitating postprocessing heat treatment. Challenges arise when using ceramics like ZrO_2_, which are prone to cracking due to thermal stresses [27]. EBM uses an electron beam in a vacuum to fuse metal powders layer by layer, offering high‐energy beams and low maintenance, but its high cost and the need for regular maintenance are significant drawbacks. Additionally, EBM produces x‐rays, which can be harmful to operators and surrounding environments [14]. *Laminated object manufacturing* (LOM) is a sheet lamination process where layers of material are bonded to create 3D objects. Using lasers and adhesive bonding agents, the layers are fused together, with adhesive agents ensuring interconnection between adjacent layers. After debinding and sintering, the final ceramic or glass ceramic parts are produced. This method allows for fast production of large, durable materials. However, it requires expertise and time, and the surface quality and dimensional stability are lower compared to other methods. Postproduction material removal is more time‐consuming, making it less suited for intricate shapes. Traditional LOM uses green ceramic tape‐cast layers cut by a CO_2_ laser and laminated with a heated roller. A variant, CAM of laminated engineering materials (CAM‐LEM), involves robotically stacking precut layers to avoid internal voids, improving layer adhesion, and reducing internal void formation [29]. Table 1 shows an integrated comparison of all the ZrO_2_ AM techniques.

**Table 1:** Integrated comparison of zirconia additive manufacturing technologies.

**AM technique**	**Mechanism**	**No. of studies**	**Key applications**	**Key advantages**	**Limitations**	**Flexural strength (MPa)**	**Surface roughness (*μ*m)**
Stereolithography (SLA)	UV laser curing of resin–ceramic mix	11	Crowns, inlays, prototypes	High accuracy, good surface finish	Support needed, expensive setup	200.14	2.06
Digital light processing (DLP)	Projected UV light curing resin layers	8	Crown frameworks, full‐arch models	Fast, accurate, better layer uniformity	Requires postprocessing, setup cost	831 ± 74	~2.0
Inkjet printing	Droplet deposition of ceramic slurry	6	Dental prosthesis frameworks	Customizable, precise, material efficient	Drying defects, ink formulation critical	~400	~3–5
Fused deposition modeling (FDM)	Extrusion of melted filament	4	Customized scaffolds	Cost‐effective, multiple materials	Weak strength, visible lines	< 300	> 5
Direct ink writing (DIW)	Extrusion of ceramic paste	3	Experimental studies only	Porosity control, scaffold design	Requires sintering, nozzle clogging	~500	~4
Binder jetting (BJ)	Binder deposited on ceramic powder	4	Porous scaffolds and restorations	Large scale, low thermal stress	High porosity, infiltration needed	200–400	> 5
Selective laser sintering (SLS)	Laser sintering of powder layers	2	Research settings only	No supports needed, complex shapes	Cracks from thermal stress	Variable	Variable
Selective laser melting (SLM)	Laser melting of metal powders	—	—	Dense parts, strong bonding	Thermal tension, limited to metals	High (metals)	< 2 (metals)
Electron beam melting (EBM)	Electron beam melting in vacuum	—	—	Strong bonds, vacuum process	High cost, x‐ray exposure	High (metals)	< 2 (metals)
Laminated object manufacturing (LOM)	Layer bonding using adhesive + laser cutting	—	—	Large objects, fast process	Low resolution, long postprocessing	Moderate	> 5

## 4. Additive Manufactured ZrO_2_ in Dental Restorations

AM in dentistry is used for various purposes, such as producing crowns, bridges, dentures, surgical guides, implants, and orthodontic devices. AM of ZrO_2_ offers several advantages and is an attractive option, particularly due to its ability to fabricate dental prostheses with reduced material waste and customized geometries [7, 30].

### 4.1. Dental Crowns and Bridges

Dental crowns and bridges are the most common dental prostheses that are studied when examining ceramic restorations with AM. 3D‐printed ZrO_2_ crowns and veneers display consistency and meet precementation standards akin to conventionally milled restorations [31]. However, support‐dependent printing technologies may affect accuracy, especially on the occlusal surface, requiring further optimization and improvement [11]. A 100% survival and success rate was observed with 3D‐printed posterior ZrO_2_ crowns, which demonstrated superior marginal fit compared to subtractive counterparts and comparable accuracy as well. Additionally, they do not harm the periodontium or contribute to the wear of opposing natural teeth [32].

### 4.2. Veneers

In minimally invasive dentistry, ZrO_2_ is one of the most used materials, and lithium disilicate is also frequently employed. Milling is one of the most widely used traditional techniques, while hot pressing is also commonly utilized. However, AM has lately drawn interest. ZrO_2_ veneers produced with DLP technology demonstrate excellent marginal fit and detail accuracy, matching or exceeding the quality of veneers made with conventional techniques [33, 34]. These veneers meet clinical standards, with average marginal gaps measuring under 0.1 mm and present reduced cement gap at the incisor edge in comparison to the milled veneers. Furthermore, they exhibit excellent load‐bearing strength and resistance to wear from tooth contact [11, 35].

### 4.3. Implants

Dental implants must meet specific criteria for tooth replacement, including the ability to function for chewing, endure occlusal forces, and resist oral fluids, biocompatibility, safety, and natural esthetics. The most commonly used materials in the construction of dental implants are titanium alloys, chrome–cobalt alloys, and ZrO_2_ [36]. ZrO_2_ is preferred over metal‐based implants for its chemical resistance, biocompatibility, and esthetic qualities. Metal components in implants can lead to loss of bone and gum recession, while ZrO_2_ implants cause fewer inflammatory reactions and less bone resorption than titanium. In vivo studies have proven that ZrO_2_ implants possess superior biocompatibility and effectively integrate with the bone, ensuring strong osseointegration. While ZrO_2_ implants have demonstrated encouraging clinical outcomes, they are not as widely used as titanium implants, primarily due to titanium′s well‐established history of reliable performance and the limited research on the clinical success of ZrO_2_ implants [37]. Advances in ceramic AM have simplified the production of ZrO_2_ implants, potentially expanding their use in dental applications. Advancements in AM and imaging technology have enabled the development of customized root‐analog implants (RAIs) tailored to the root morphology of extracted teeth. These RAIs align precisely with postextraction sockets, improving primary stability, reducing bone loss, and simplifying insertion [38]. DMLS implants offer high fatigue strength, good corrosion resistance, and fracture strength exceeding 1200 N for dimensions of 3.5 by 16 mm. However, achieving a precise fit between the implant and abutment in two‐stage dental implants is challenging due to surface accuracy limitations, which can be addressed by machining the connection structure [39].

Custom‐designed 3D‐printed implants fabricated using DLP technology exhibit precise dimensions, with a root mean square error of 0.1 mm and a flexural strength of 943.2 MPa, comparable to traditionally milled ZrO_2_ (800–1000 MPa). The surface roughness is measured at 1.59 *μ*m. SEM analysis reveals microporosities with interlinked spaces spanning from 0.196 to 3.3 *μ*m, along with visible cracks. These defects are attributed to the sintering process or the inadequate dispersion of ceramic particles in the slurry. Thus, optimizing 3D printing parameters could improve the microstructural quality of printed implants [40]. On the evaluation of the mechanical profile and microstructure of sintered implants produced using a 3D slurry printing system with a dual‐stage sintering process, the green body exhibited low flexural strength (20.41 ± 3.8 MPa) and hardness (0.12 GPa), while the sintered specimens possessed significantly enhanced properties, including a flexural strength of 632.1 ± 72.5 MPa and a hardness of 14.72 GPa. The dental implant model was refined to reduce micromotion; however, further improvements in the accuracy of the final parts are still required [41]. The optical scans of the DLP ZrO_2_ RAI show notable differences from the original tooth, particularly near the apical foramen, with a maximum deviation of 0.86 mm. When limiting deviations to 0.5 mm, 1.55% and 4.86% of the 3D‐printed RAI surfaces exceed this threshold. Despite these deviations, current technology makes it feasible to 3D print a custom RAI in ZrO_2_ [42].

### 4.4. Bone Regeneration

ZrO_2_ bioceramics have experienced great success in the dental field, especially demonstrated in dental posts, teeth, and crowns. This has inspired researchers to explore their properties for bone regeneration applications [43]. The prospect of 3D printing, particularly in the construction of ZrO_2_ scaffolds, is promising. Biomedical engineers have focused on techniques like FDM, DLP, DIW, and SLS for ZrO_2_ scaffold development. The process of FDM includes ZrO_2_ ceramics that are blended with polymers such as polycaprolactone to create a regular grid scaffold. The addition of biopolymers in FDM‐produced ZrO_2_ scaffolds enhances mechanical support and bioactivity, mimicking the bone tissue environment [44].

Direct write printing (DWP), an extrusion‐based AM technique, produces high‐porosity ZrO_2_ scaffolds, requiring hydroxyapatite/fluorapatite coatings to enhance bioactivity. DLP technology offers high accuracy and speed, using ultraviolet light to solidify ZrO_2_ suspensions. Heat treatment in a high‐temperature vacuum furnace is crucial to prevent cracks in the final ZrO_2_ scaffolds. SLS, while widely used for calcium‐based bioceramics, faces limitations in ZrO_2_ due to low concentration; ZrO_2_ is often blended with other bioactive materials. Additionally, electrospinning ZrO_2_‐based scaffolds replicates the nano‐to‐microscale bone tissue configuration, enhancing their durability against bone tissue loads compared to traditional fragile scaffolds [27]. Creating ZrO_2_ scaffolds using DWP involves depositing a 70% solid content water‐based ZrO_2_ ink layer by layer using a tiny nozzle. These scaffolds exhibited greater compressive strength and facilitate the proliferation of HCT116 cells around it. Therefore, AM‐DWP technology enables the precise manufacturing of ZrO_2_ scaffolds with controlled porosity, making them suitable for advanced bone tissue engineering applications [45].

### 4.5. Esthetic Restorations

Recent advances in ZrO_2_ materials have significantly improved their suitability for esthetic restorations. Multilayered partially stabilized ZrO_2_s, such as 4Y‐TZP and 5Y‐TZP, provide higher translucency and natural gradient shading due to increased cubic phase content and refined grain structures, achieving an enamel‐like appearance without veneering ceramics [46, 47]. While 4Y‐TZP offers a favorable balance between strength (600–800 MPa) and esthetics, 5Y‐TZP further enhances translucency but has lower fracture resistance, requiring careful case selection [48]. Surface treatments including glazing and polishing improve gloss and color stability, making modern ZrO_2_ a versatile option for anterior crowns and veneers [49].

Table 2 provides a comprehensive compilation of recent studies with AM of ZrO_2_, highlighting the key findings over the years, along with a comparison of AM versus SM techniques.

**Table 2:** Compilation of recent studies with additive manufacturing of ZrO_2_.

	**Author and year**		**Methodology**	**Key findings**	**AM vs. SM comparison**	**Impression**
1.	Revilla‐Leon et al. [16] (2020)	Assessed and compared the flexural strength and Weibull characteristics of ZrO_2_ produced through milling and AM processes	Milled and AM samples tested pre‐ and postaging simulation	Milled ZrO_2_ group demonstrated significantly higher fracture resistance and flexural strength values compared to the AM ZrO_2_ group in preaging test conditions Aging reduced strength in both groups	Milled ZrO_2_ proved to be superior pre‐ and postaging	The manufacturing process significantly impacted the flexural strength of the tested ZrO_2_. Mastication simulation, used to accelerate artificial aging, led to markedly reduce flexural strength values for both ZrO_2_ types
2	Su et al. [50] (2020)	Assess pristine vs. recycled ZrO_2_ stereolithography	SLA with 20 *μ*m (pristine) and 40 *μ*m (recycled) powder; tested density, hardness, strength	Pristine: > 99% density, 1057 MPa strength; recycled: ~90% density, lower strength	Pristine AM meets ISO standards. The recycled component underperforms	Pristine AM suitable for load bearing; recycled optimum for nonload applications
3	Nakai et al. [51] (2021)	Assess microstructure and strength of AM vs. SM 3Y‐TZP	Compared milled and AM sintered specimens	The additively manufactured LithaCon 3Y 230 showed markedly lower biaxial flexural strength and higher porosity than ATZ	SM was found to be better than AM in terms of strength	3Y‐TZP via AM exhibited comparable microstructure, crystallography, and flexural strength to ZrO_2_ SM highlighting its potential suitability for dental implants
4	Sakthiabirami et al. [52] (2021)	Evaluate 3D hybrid ZrO_2_ biopolymer constructs for bone engineering	AM fabrication of porous hybrid ZrO_2_, compressive strength, and bioactivity tests	Biopolymer‐infused frameworks improved compressive strength by 20%, enhanced osteogenic differentiation, and demonstrated superior structural performance compared to HA/glass surfaced glass‐infiltrated zirconia (HZrO_2_) framework	AM constructs demonstrated significant functional improvements over conventional HA/glass coatings	Hybrid AM ZrO_2_ templates are promising for load‐bearing bone regeneration applications
5	Moon et al. [53] (2021)	Assessed the results of repeated firing on the surface profile, *S. mutans* viability, and optical properties of dental ZrO_2_ during the AM process after sintering	Additive firing cycles with surface roughness, translucency, and *S. mutans* viability assessment	Additive firing reduced roughness, contact angle, and bacterial viability without affecting optical properties; repeated firings had no additional effects	AM firing demonstrated improved antibacterial properties and surface characteristics compared to single firing	Additive firing post‐AM may prevent secondary caries while preserving esthetics
6	Kim et al. [54] (2022)	Assessed the accuracy of the intaglio surface, volume of antagonist wear, and fracture load in AM vs. SM	Tested single‐unit ZrO_2_ crowns for surface deviations, wear volume, and fracture resistance	Intaglio surface deviations < 0.05 mm for all methods; no notable wear differences; both fabrication and chewing simulation reduced fracture resistance	AM crowns showed to have clinically acceptable accuracy and wear comparable to SM, but fracture load was reduced	AM is feasible for single crowns but fracture strength requires optimization
7	Jang et al. [14] (2022)	Investigated the physical and mechanical properties of silane‐modified ZrO_2_ volumetric in DLP‐based AM	Tested hardness, strength, and density with silane coupling agent	~6% increase in hardness and strength; reduced ZrO_2_ particle size improved density	Silane‐modified AM samples demonstrated improved mechanical properties over unmodified AM	Silane‐modified ZrO_2_ particles through AM exhibited beneficial effects on the physical properties
8	Moon et al. [55] (2022)	Evaluated the manufacturing accuracy and bond strength between porcelain and ZrO_2_ via AM and SM	Measured marginal accuracy and bond strength between porcelain and ZrO_2_	AM samples had marginal accuracy comparable to SM; bond strength higher in AM specimens	AM superior in bond strength and similar in dimensional accuracy compared to SM	AM technology shows considerable promise for use in dentistry, especially bonding applications
9	Roser et al. [56] (2022)	Evaluated osteoblast behavior on AM and milled 3 mol% 3Y‐TZP ZrO_2_	Surface roughness and biocompatibility assessment	AM‐unmodified ZrO_2_ presented greatest surface roughness, enhancing osteoblast adhesion No differences between groups observed in relation to cell adhesion and proliferation	AM surfaces demonstrated higher roughness and favorable cell response compared to SM	AM supports improved osseointegration potential, though roughness control is necessary
10	Kang et al. [57] (2022)	Examined how UV absorbers impact the dimensional accuracy of ZrO_2_ samples made with AM using a DLP	Tested expansion and precision with varying UV absorber levels	UV absorbers were found to have decreased the geometric expansion from ~12% to ~2%; however, inclusion had minimal impact on cure depth	The use of UV absorber enhanced the AM stability compared to unmodified DLP	UV absorbers can be incorporated for enhanced precision in 3D multilayer ceramic restorations
11	Tan et al. [58] (2022)	Compared the impact of accelerated aging on the physical and biological characteristics of ZrO_2_ produced using DLP and traditional SM	DLP and SM samples were tested for microstructure, phase transformation, and cellular response	DLP ZrO_2_ exhibited a higher initial cubic phase content and a faster phase transformation rate compared to SM‐fabricated ZrO_2_ Both types showed similar biological performance pre‐ and postaging, with only minor differences in cell alignment and morphology	DLP showed similar biocompatibility but faster phase changes compared to SM	Considered suitable for use as an implant abutment material, keeping in mind the aging effects
12	Frackiewicz et al. [59] (2023)	Appraised the mechanical and functional characteristics of 3D printing vs. conventional dental milled ZrO_2_ ceramics	Compared the surface analysis and mechanical parameters between both the groups	Both ceramics exhibited similar mechanical values with no statistically significant differences Geometric arrangement was identical. AM group had slight surface cracks	AM ceramics demonstrated comparable mechanical and surface properties to SM	Strong potential for integration into clinical practice
13	Yoo et al. [60] (2023)	Compared the mechanical properties of AM vs. SM ZrO_2_	Flexural strength, Vickers hardness, phase content, surface roughness	The flexural strength and Vickers hardness of AM ZrO_2_ fabricated were slightly lower than those of SM ZrO_2_ but within clinically acceptable ranges Higher monoclinic phase content, flexural strength, and surface roughness of the AM group due to air abrasion	AM samples comparable in overall performance to SM though surface treatment increased roughness	AM restorations are clinically acceptable, but surface finishing protocols require optimization
14	Gallicchio et al. [61] (2023)	Examined new ZrO_2_ nanoparticulate light‐activated and AM fabrication methods	Fabrication and characterization of nanoparticle‐reinforced composites	Mechanical characteristics modified by altering the form and amount of filler used. Heavily reinforced composites successfully produced via AM (particularly 3D DLP)	ZrO_2_ nanoparticles enhance mechanical properties Outperforming traditional methods for specific purposes	AM holds promise for advanced reinforced composites with tailored mechanical profiles via 3D fabrication
15	Frackiewicz et al. [62] (2024)	Evaluate the biofilm formation and microorganism attachment in AM and SM techniques	Measured biofilm deposition by various oral pathogens on fabricated surfaces	No variations were noted in the amount of biofilm formed by *Streptococcus mutans*, *Pseudomonas aeruginosa*, *Enterococcus faecalis*, and *Staphylococcus aureus* Slightly higher *Candida albicans* on AM samples but not statistically significant	AM surfaces had comparable microbial performance to SM as it did not increase biofilm formation	AM zirconia can be safely used without added biofilm risk compared to conventional milling
16	Darbandi and Amin [63] (2024)	Assessed the efficacy of functionalized loading of ZrO_2_ nanoparticles and Ag‐nanotube composites in 3D printing	Tested using different nanoparticle loadings and by measuring mechanical properties	Adding 4% ZrO_2_ + 5% HNC/Ag notably improved the flexural strength, fracture toughness, and flexural modulus when compared to the control group 16% ZrO_2_ + 5% HNC/Ag presented notably higher hardness compared to other groups	Nanoparticle incorporation significantly enhanced AM composites over unmodified resins	Functionalized nanoparticles improve strength and toughness of 3D‐printed restorations
17	Cho et al. [64] (2024)	Compared the trueness, physical, and surface properties of five different types of ZrO_2_ crowns using AM method against those fabricated using SM	Evaluated trueness, void presence, and surface roughness across five AM groups	The DLP group was found to have consistent trueness; all AM groups had voids and higher surface roughness AM showed comparable trueness to SM but more surface imperfections	AM crowns showed higher surface roughness and voids, requiring refinement in processing	AM is promising but needs optimization of printing and finishing protocols to match SM surface quality

## 5. Challenges in AM of ZrO_2_

Although the AM technique has been found to have several advantages and good properties, however, when compared to SM, it still holds some drawbacks that hinder its potential for a broader use in clinical dentistry.

### 5.1. Mechanical Strength Limitations

As shown by various studies, many AM techniques are still not comparable to match the flexural strength and fracture toughness achievable through conventional milling. Although SLA and DLP have the potential to produce high‐resolution parts, they have a major drawback of residual porosity due to incomplete polymer burnout during sintering, which leads to the weakening of the final ceramic structure [65]. It is also seen that DLP ZrO_2_ crowns often have lower flexural strength (~800 MPa) as compared to their milled counterparts (> 1000 MPa), which is unfavorable for thin‐wall applications like veneers. FDM‐based ZrO_2_ filaments are prone to layer delamination due to incomplete interlayer fusion and binder removal defects [66].

### 5.2. Dimensional Instability and Shrinkage

SLA holds a disadvantage of postcuring shrinkage during sintering, which may reach up to 20%–25%, thereby making precision fit challenging, especially in the case of multiunit restorations [67]. Rapid heating and cooling during powder bed fusion processes induce thermal gradients and multiple residual stresses, leading to parts distortion or microcracks [68]. Drying and sintering cause anisotropic shrinkage, leading to warping or loss of marginal fit, as seen during the BJ process. The use of UV absorbers or higher solid loading is shown to increase slurry viscosity and printing complexity [67].

### 5.3. High Porosity and Internal Defects

Porosity is inherent in many AM processes and directly compromises strength, translucency, and biocompatibility. Layer interfaces can trap interstitial voids and binder residues, causing crack initiation [69]. In order to mitigate porosity, additional processing steps like vacuum infiltration or isostatic pressing are often employed, which may increase the processing time and cost of the final product [65].

### 5.4. Economic Considerations and Production Time

Industrial AM equipment for ceramics (e.g., DLP printers and SLS systems) can cost $100,000–$500,000, significantly more than desktop milling units [70]. AM fabrications require multistage processes (involving printing, debinding, sintering, infiltration, and finishing) which usually take significantly longer than subtractive milling. Though AM is shown to have promising results for custom‐made crowns, however, mass production of routine crowns in dentistry is not cost‐efficient when compared to conventional milling [47].

### 5.5. Barrier to Clinical Translation

Variability in porosity, shrinkage, and residual contamination complicates ISO and FDA certification. Reproducibility studies are limited compared to mature SM workflows [71]. Surface and mechanical limitations, regulatory hurdles, and lack of long‐term data delay widespread clinical acceptance. [65]

## 6. Emerging Technologies and Advancements in ZrO_2_ AM

Recent studies have introduced several promising developments to address traditional limitations of ZrO_2_ AM:

• Nanostructured ZrO_2_ and ZTA composites improve fracture toughness and wear resistance, offering enhanced mechanical performance compared to conventional ZrO_2_ ceramics [46, 72].
• Gel‐casting‐based AM and hybrid AM‐subtractive workflows allow finer control over particle dispersion, shrinkage compensation, and microstructure densification [67, 73].
• DLP continues to evolve with optimized photopolymer suspensions, achieving higher density and translucency [74, 75].
• Surface functionalization techniques, such as incorporating silane coupling agents or bioactive coatings, improve adhesion to resin cements and biological tissues, expanding clinical applicability [71, 76].

Additionally, recent studies comparing fracture loads of translucent ZrO_2_ crowns demonstrate promising mechanical stability under functional loads [77].

## 7. Future Perspectives and Targeted Research Directions

ZrO_2_ is a crucial bioceramic, which is extensively researched for dental and biomedical applications. However, several limitations hinder AM technologies in creating ZrO_2_‐based ceramics effectively. While various AM techniques exist, each has specific advantages and limitations.

### 7.1. Feedstock and Material Development

The feedstock preparation, especially for ZrO_2_ ceramics, remains a significant hurdle, affecting nozzle clogging and print quality [73, 78]. Efforts are needed to create stable suspensions, control rheology, and optimize viscoelastic properties [74, 78]. The study of particle size distribution is crucial for improving density, flowability, and minimizing shrinkage in different AM methods [6, 79]. It is also essential to explore the use of dopants or additives that can enhance densification and reduce porosity during sintering [79].

### 7.2. Process Optimization

To improve dimensional accuracy and reproducibility, it is pertinent to control standardized process control strategies [78]. Mechanical strength can be improved by incorporating pressure‐assisted methods to reduce internal porosities. Graded properties of natural teeth can be replicated better by the introduction of biomimetic design strategies [27].

### 7.3. Clinical Validation

To clinically validate the strength and potential of AM ZrO_2_, it is important to conduct long‐term in vitro studies stimulating the oral environment such as artificial saliva baths, thermal cycling, and mechanical fatigue stimulators. Long‐term clinical trials should also be developed to validate AM ZrO_2_ durability in order to assess phase transformation, microleakage, wear resistance, and microbial adhesion over extended periods [49, 79].

### 7.4. Mechanical and Surface Property Enhancement

Future AM research should focus on developing low‐porosity ZrO_2_ slurries for enhanced mechanical properties, microstructure, and dimensional accuracy to bridge the gap with traditional methods [68, 78, 79].

### 7.5. Integration of Digital Technologies and Future Perspectives

Along with all this, there is also a scope of integrating AI for real‐time printing parameter optimization [80]. Predictive modeling tools can be utilized to simulate shrinkage and deformation before printing to establish a more reliable AM workflow [81].

## 8. Limitations

While this review offers a thorough synthesis of AM technologies in ZrO_2_ dentistry, there are certain limitations associated with this research. Firstly, this review primarily considered articles published in the English language and indexed journals, potentially excluding relevant data from non‐English or gray literature sources. While this review paper summarizes all the available in vitro and short‐term in vivo studies, there is still a huge scarcity of long‐term clinical evidence (≥ 5 years) evaluating the survival and performance of AM‐fabricated ZrO_2_ restorations in real patients. AM technologies and material formulations are evolving rapidly. Therefore, some findings may become outdated as new techniques and materials emerge.

## 9. Conclusion

In the field of dentistry, especially in restorative and implant dentistry, AM of ZrO_2_ holds great potential. Technologies like DLP and STL have become particularly important due to their widespread use in creating ZrO_2_ dental restorations. These methods offer significant advantages, including the ability to produce highly precise and customized dental solutions. However, improvements are still needed in printer design, material formulations, and printing settings to make the most of AM ZrO_2_. More research is crucial, especially to better understand aspects such as optical properties, biocompatibility, residual resin residues, and bonding compatibility with porcelain layering techniques before fully integrating this technology into clinical practice. Despite these challenges, AM′s ability to produce ZrO_2_ with a range of desirable properties makes it a promising option for the future of restorative, implant, and regenerative dentistry. As technology and materials continue to improve, additive ZrO_2_ is set to become an essential part of modern dental care, providing more personalized and efficient treatment options for patients.

**Nomenclature**
AM: additive manufacturing
BJ: binder jetting
CAD/CAM: computer‐aided designing/computer‐aided manufacturing
CAM‐LEM: CAM of laminated engineering materials
CNC: computer numeric controlled
DLP: digital light processing
DMD: digital micromirror device
DMLS: direct metal laser sintering
DIW: direct ink writing
EBM: electron beam melting
FDM: fused deposition modeling
LOM: laminated object manufacturing
RIA: root‐analog implants
SLA: stereolithography
SLS: selective laser sintering
SLM: selective laser melting
SM: subtractive manufacturing
3Y‐TZP: yttrium tetragonal ZrO_2_ polycrystals
ZTA: zirconia‐toughened alumina
ZLS: ZrO_2_‐containing lithium silicate
ZrO_2_
: zirconia

## Conflicts of Interest

The authors declare no conflicts of interest.

## Author Contributions

Gunjan Singh Aswal: conceptualization, methodology, writing—original draft, and supervision; Renu Rawat: resources, investigation, and project administration; Reisha Rafeek: resources, investigation, and project administration; Dhara Dwivedi: data curation, methodology, and writing—original draft; Nitin Prabhakar: validation and writing—review and editing; Madan Mohan Gupta: validation and writing—review and editing

## Funding

No funding was received for this manuscript.

## Data Availability

Data sharing is not applicable as no new data were generated or the article describes entirely theoretical research.
