# Correction to “Additive Zirconia in Dentistry: Techniques, Trends, and Future Perspectives”

**DOI:** 10.1155/bmri/9893132

**Published:** 2026-02-02

G. S. Aswal, R. Rawat, R. Rafeek, D. Dwivedi, N. Prabhakar, and M. M. Gupta, “Additive Zirconia in Dentistry: Techniques, Trends, and Future Perspectives,” *BioMed Research International*, 2025, https://doi.org/10.1155/bmri/6602281

In the abovementioned article, there was an error in Reference 7: An incorrect DOI was included. The correct reference is as follows:

G. S. Aswal, R. Rawat, D. Dwivedi, N. Prabhakar, and V. Kumar, “Clinical Outcomes of CAD/CAM (Lithium Disilicate and Zirconia) Based and Conventional Full Crowns and Fixed Partial Dentures: A Systematic Review and Meta‐Analysis,” *Cureus* 15, no. 4 (2023): e37888, https://doi.org/10.7759/cureus.37888.

The online version of this article has been corrected accordingly.

We apologize for these errors.

